# Investigating the impact of a novel movement behavior program for refugee youths in Canada: the IPLAY program

**DOI:** 10.3389/fspor.2025.1668416

**Published:** 2025-12-05

**Authors:** Taylor Rowe, Jeffrey D. Graham, Sujane Kandasamy, Jennifer Konopaki, Amanda Koyama, Matthew Y. W. Kwan

**Affiliations:** 1 Department of Health Sciences, Brock University, St. Catharines, ON, Canada; 2 Department of Child and Youth Studies, Brock University, St. Catharines, ON, Canada; 3 WinSport, Calgary, AB, Canada; 4 Calgary Catholic Immigration Society, Calgary, AB, Canada; 5 Department of Community Health Sciences, University of Calgary, Calgary, AB, Canada; 6 School of Movement and Nutrition Sciences, University of Queensland, Brisbane, QLD, Australia

**Keywords:** novel movement program, physical literacy, newcomer, refugee, settlement

## Abstract

**Introduction:**

Physical literacy (PL) is considered to be a multidimensional concept that includes the core domains of movement competence, confidence, motivation, and knowledge and understanding related to regular participation in physical activity (PA). The Immigrant-focused Physical Literacy for Youth (IPLAY) program was an 8-week initiative designed for newcomer youth that took place in the Fall of 2022 and Winter of 2023. This study examined the potential impact of the IPLAY program on PL development among newcomer youth.

**Methods:**

Participants for the current study included a total of 38 refugee youths (*M*_age_ = 14.84, *n* = 22 males and 16 females) between the ages of 12–17 years and recent arrivals to Canada (76% having moved to Canada within the past 12 months). Measures of PL included an observed assessment of movement competence (PLAY*basic*), along with PLAY*self* as self-reported assessments of confidence, motivation and knowledge and understanding.

**Results:**

Overall, results found that PA levels (*p* = 0.03) and overall movement competence (*p* = <.001) significantly increased by the end of the intervention. Whilst there were increases in other domains of PL, they were not found to be statistically significant.

**Discussion:**

Overall, the present study is encouraging, providing initial support for community-based programs to aid in the PL development among newcomer youths to Canada. Future trials with larger and more diverse samples of newcomers are needed to further demonstrate the efficacy of PL-based programs such as IPLAY.

## Introduction

Regular engagement in physical activity (PA) confers a myriad of social, developmental, and health benefits for children and youth (1, 2). This includes but is not limited to greater social-emotional development, cognitive functioning, mental wellbeing, and chronic disease prevention (1, 3). Despite the many benefits, only one-third of Canadian children and youth are meeting PA recommendations of 60 minutes of moderate-to-vigorous intensity PA engagement each day (4). More alarming is that newcomer children and youth have been consistently found to be even less active when compared to their peers born in Canada (5).

Canada has traditionally had one of the highest acceptance rates of immigrants; however, recent immigration policies have seen the number of newcomers rapidly rise in recent years (6). Interestingly, many newcomers tend to arrive with good health status; however, their health status generally declines during their transition into a new country (7–9, 28). This may be in part due to negative changes in PA patterns or decreased PA behaviors (10–12). Current literature suggests that newcomer children and youth will face challenges to their PA participation, such as language barriers, cultural differences, and/or lack of equitable access due to financial constraints (8, 11, 13). Given the rising number of young newcomers to countries such as Canada over the next several years, it is critical that we better understand effective strategies to help this traditionally marginalized population maintain physically active lifestyles.

In recent years, some experts have argued that many children and youth lack the necessary fundamental movement skills, motivation, positive feelings toward, and knowledge and understanding to sustain physically active lifestyles. These represent the core domains of physical literacy (PL), a multifaceted concept that essentially unites several previously siloed theoretical paradigms (14, 15). Movement competence represents the motor skills required for movements across a wide range of contexts, including land, air, or water (16, 17). Motivation is impacted by positive affect, often expressed in terms of fun and enjoyment, while confidence to move and participate is also central to the concept (14). The final domain within PL is the knowledge and/or understanding of PA behaviors in terms of its benefits and opportunities (14, 16). Importantly, there is robust literature indicating that each of the core domains of PL have been found to be consistent correlates or determinants of PA behaviors (18), thus intervention efforts that specifically target PL through broader movement skill development using fun activities and conducted within supportive environments may be particularly beneficial for populations such as recent newcomers to Canada.

While the existing literature has demonstrated some successes in helping young people develop PL through broader holistic programs targeting participants’ competence, confidence, motivation and knowledge and understanding (19), no intervention research has focused on the newcomer population. Therefore, the objective of the current study was to evaluate the potential efficacy of a pilot PL-based novel movement program entitled the *Immigrant-focused Physical Literacy for Youth* (IPLAY). Specifically, it was hypothesized that this novel movement intervention program that intentionally targets each PL domain would positively impact overall PL development among refugee youths, including movement competence, confidence, motivation and knowledge and understanding. Additionally, it was expected that there would be improvements in participants’ overall PA behaviors.

## Materials and methods

### Study design and participants

Applying a quasi-experimental study design, 68 refugee youths were initially recruited into this subsidized program. In partnership with a local newcomer service agency (the Calgary Catholic Immigration Society), participants were recruited from within the community in Fall 2022 and Winter 2023 and were selected based on their interest in participating in a multi-sport and activity program. Using a multipronged recruitment strategy, posters and promotional materials as well as a study letter of information were sent via e-mail to the refugee community. Interested participants then contacted a member of the local newcomer service agency or research team to sign up for the program.

Due to a variety of logistical or unknown issues (i.e., difficulties with transportation (*n* = 2), conflicting schedules with other subsequent activities (*n* = 5), unknown reasons for drop-out (*n* = 14) and absence for regular participants during either the first or final sessions where data collection took place (*n* = 9), complete data was ascertained for a total of 38 participants (*M*_age_ = 14.84, *n* = 22 males). This included participants that took part in either the Fall 2022 (*n* = 21) or the Winter 2023 (*n* = 17) programs. Baseline data collection took place during week 1 of both Fall and Winter sessions. Post-intervention data collection took place during the final week of the program. Both IPLAY programs were delivered at a local recreational facility (WinSport), and bus transportation was arranged for youths living in different regions of the city. With the help of the newcomer agency, parental/guardian consent for study participation and participant assent were collected prior to or during the baseline assessments. Participants received $25 gift cards for participation for completion of baseline and follow-up assessments. All study protocols were approved by the Brock Research Ethics Board #21-295.

### The IPLAY intervention

The IPLAY intervention was designed as an 8-week pilot PL–based novel movement intervention. Each week, youths engaged in 90-minute sessions that were uniquely designed to have participants practice movements in different ways (e.g., locomotion, balance, upper and lower limb control) and within different contexts (e.g., on land, air, or ice). Each session was intentionally designed to build competencies in movement, with intervention leaders aiming to create an environment that helps youth build confidence, motivation, and knowledge and understanding. Two trained program leaders delivered each program session, with a program manual that specified how each activity would correspond with specific movement domains (i.e., motor competence) and participant learning objectives (i.e., knowledge and understanding). In addition, two youth program facilitators (i.e., university students) from the University of Calgary attended each session to help with translation, while participating with the youth participants in the activities. Prior to each session, the program leaders would meet to identify specifically how each session could be structured to be a fun and supportive group environment, develop communication strategies for clear instructions to the various activities and games, and determine how strengths in competencies for each participant would be highlighted (i.e., targeting confidence and motivation). As an example, there was one week that focused on wheelchair basketball, where participants learned about adapted sports, engaged in various drills learning to wheel around a basketball court, pass and shoot from a seated position, and to engage in some friendly relay races and basketball games. Program leaders would encourage and motivate the youth participants in trying these new activities and having fun with it. Other weekly games and activities that participants engaged included: Gaga ball (i.e., an adapted form of dodgeball within an octagon space), Kin-ball (i.e., a Canadian-developed game utilizing giant inflatable balls), human curling (i,e., teams pushing inflated inner tubes on face to targets), and ice luge (i.e., sliding activity on an indoor luge start track).

### Study measures

#### Physical literacy

Overall and specific domains of PL were assessed using the PLAY*basic* tool and a modified version of the PLAY*self* tool. The same assessments were completed by all participants at baseline (pre-intervention) and following up (post-intervention). Consistent with previous research, a composite PL score was calculated using standardized scores of all PL measures (20). Standardized z-scores were calculated separately based on the total scores of the PLAY*basic*, confidence, motivation, and knowledge and understanding sub-domains. Scores were subsequently summed, with higher values reflecting greater PL.

#### Movement competence

Movement competence was assessed using the PLAY*basic* tool. PLAY*basic* is a simplified and validated version of the PLAY*fun* tool, used for individuals aged 7+, and is composed of five tasks that measure an individual's physical abilities (21). PLAY*basic* includes five movement tasks divided into five subdomains: (1) Running (i.e., run there and back), (2) Locomotor (i.e., hop on one leg), (3) Object control- upper body (i.e., overhand throw), (4) Object control- lower body (i.e., kicking a soccer ball), and (5) Balance, stability and body control (i.e., backward (toe-to-heel) walk. The assessments were carried out by trained researchers. In some cases, the researchers were also accompanied by translators to interpret instructions into the participants’ native language. Each task was evaluated using a 100 mm visual analog scale (VAS). The scale is divided into four categories: initial (0–25 mm), emerging (25–50 mm), competent (50–75 mm), and proficient (75–100 mm). The total score was calculated by averaging the 5 tasks, and the subdomain scores were calculated by averaging the tasks within each domain. Higher scores are indicative of greater movement competence among participants.

#### Perceived confidence

Confidence was assessed using 1-item from the PLAY*self* tool: “*I’m confident when doing physical activities*”. Participants rated this on a 4-point scale from 1 (*Not true at all*) to 4 (*Very true)*. Additionally, perceived confidence was assessed using 6-items from the PLAY*self* tool, which included (1) “*It doesn’t take me long to learn new skills, sports or activities*”, (2) “*I think I have enough skills to participate in all the sport and activities I want”*, (3) “*I think I can take part in any sport/physical activity I choose”*, (4) “*My body allows me to participate in any activity I choose”*, (5) “*I'm usually the best in my class at doing an activity”*, and (6) “*I don’t really need to practice my skills, I’m naturally good”*. Each item was rated on a 4-point scale ranging from 1 (*Not true at all*) to 4 (*Very true)* (Cronbach *α*'s = 0.68–0.85).

#### Motivation

Motivation was assessed using 3-items from PLAY*self* tool, which included (1) “*I think being active makes me happier”*, (2) “*I worry about trying a new sport or activity”*, and (3) “*I can’t wait to try new activities or sports”*. Each item was rated on a 4-point scale from 1 (*Not true at all*) to 4 (*Very true)*. Item two was reverse-scored so that higher scores reflected a higher level of motivation, and total scores were averaged across the three questions (Cronbach *α*'s = 0.52–0.58).

#### Knowledge and understanding

Knowledge and understanding were assessed using three items from PLAY*self*, including: (a) *It is important to be active every day*, (b) *Being active will increase your energy level*s, and (c) *Being active will make you feel healthy*. Each item was rated on a 4-point scale from 1 (*Not true at all*) to 4 (*Very true)* and total scores were averaged across the three questions (Cronbach *α*'s = 0.70–0.74).

#### Physical activity

PA was assessed using 2-items from the Physical Activity Questionnaire for Older Children [PAQ-C: Crocker et al. (22)]. The first item was “*Thinking back during your physical education classes (gym class), how often were you very active?*” and was rated on a 5-point scale from 0 (“*I don't do physical education*” to 4 (*Always*). The second item was “*During the past 7 days on how many days were you physically active for a total of at least 60 minutes per day?*” and was rated on an 8-point scale from 0 (*0 days*) to 7 (*7 days)*. Each item was analyzed separately.

### Data analysis

Descriptive statistics were first computed. A one-way ANOVA was conducted to examine to determine if there were differences in the demographics between those that sustained participation in the program to those that dropped out. Paired-Samples *t*-tests were done to examine changes in mean scores from baseline to follow-up. All statistical analyses were conducted using SPSS 25 (23).

## Results

### Demographics and group characteristics

Descriptive statistics for demographics and group characteristics are shown in Table 1. Data analysis was conducted with the 38 participants with complete data. One-way ANOVA analyses indicated that there were no differences (all *p*'s < 0.05) in demographic variables (age and gender) and PL variables between those with only baseline assessments to the sample with complete data used in this present study. Participants were predominantly refugees from Afghanistan (92.1%) who had been in Canada for less than 12 months (76.3%) and were between the ages of 12 and 17 years. Of note, recruitment was focused on youth 14-17 years; however, several younger siblings asked to participate.

**Table 1:** Demographics and group characteristics.

Demographics	Total (*N* = 38)	
	*M*	*SD*
Age	14.84	(1.63)
	*n*	*%*
Gender		
Female	16	42.1
Male	22	57.9
Time in Canada		
<1 year	29	76.3
1 year	6	15.8
Not answered	3	7.9
Country of Origin		
Afghanistan	35	92.1
Afghanistan–Takistan	1	2.6
Tajikisatan	1	2.6
Turkey	1	2.6

*M*, mean; *SD*, standard deviation; %, percentage.

### Physical literacy

Overall, although results found that PL composite scores increased over time, these increases were not statistically significant (*t* *=* −0.31, *p* *=* 0.76, Cohen's *d* = 0.05). Results, however, showed statistically significant improvements in overall movement competence (PLAY*basic* total score*, t* *=* −6.11, *p* = <.001, Cohen's *d* = 0.99), and across the subdomain scores for running (*t* *=* −8.02, *p* < 0.001, Cohen's *d* = 1.30), locomotor (*t* *=* −6.48, *p* < 0.001, Cohen's *d* = 1.05), objective control-upper (*t* *=* −4.05, *p* < 0.001, Cohen's *d* = 0.66), object control-lower (*t* *=* −3.60, *p* = 0.001, Cohen's *d* = 0.58), and balance, stability and body control (*t* *=* −5.03, *p* < 0.001, Cohen's *d* = 0.82). However, no significant differences were observed for perceived competence (*t* *=* −0.71, *p* = 0.49, Cohen's *d* = 0.12), confidence (*t* *=* −1.89, *p* = 0.07, Cohen's *d* = 0.31), motivation (*t* *=* −0.73, *p* = 0.47 Cohen's *d* = 0.12), and knowledge and understanding (*t* *=* −1.46, *p* = 0.15, Cohen's *d* = 0.24). Descriptive statistics and *t*-test summaries for all PL outcomes are shown in Table 2.

**Table 2:** Physical literacy and physical activity scores from baseline to post-intervention.

Variable	Baseline	Post-test	*t-value*	*p*-value
	*M* (*SD*)	*M* (*SD*)		
PLAY*basic* total	55.42 (3.76)	62.45 (7.70)	−6.11	<.001
PLAY*basic* running	55.26 (5.14)	63.24 (7.39)	−8.02	<.001
PLAY*basic* locomotor	55.59 (3.64)	62.34 (7.41)	−6.48	<.001
PLAY*basic* OC-upper	56.55 (8.81)	63.39 (10.03)	−4.05	<.001
PLAY*basic* OC-lower	54.11 (9.18)	62.08 (10.47)	−3.60	.001
PLAY*basic* balance	55.76 (5.03)	61.21 (6.74)	−5.03	<.001
Confidence	3.13 (0.70)	3.39 (0.72)	−1.89	.07
Perceived competence	3.06 (0.62)	3.15 (0.56)	−.71	.36
Motivation	3.08 (0.69)	3.18 (0.55)	−.73	.47
Knowledge and understanding	3.28 (0.71)	3.49 (0.57)	−1.46	.15
PL composite score	−.11 (3.86)	.07 (3.11)	−0.31	.76
PA during PE	3.16 (1.03)	3.11 (1.03)	0.26	.80
Daily PA of 60 min	3.95 (2.62)	4.97 (2.12)	−2.31	.03

*M*, mean; *SD*, standard deviation; OC, object control; PA, physical activity; min, minutes.

### Physical activity

Results from the paired samples *t*-tests showed significant improvements in self-reported PA behaviors (*t* *=* −4.97, *p* = 0.03, Cohen's *d* = 0.37). There were no differences found in PA participation during physical education classes (*t* *=* 0.26, *p* = 0.80, Cohen's *d* = 0.04). Descriptive statistics and *t*-test summaries are shown in Table 2.

## Discussion

Overall, results from this study provide preliminary evidence supporting the use of community-based PL-based interventions to support the PA behaviors and PL development among refugees to Canada. In particular, findings indicate that this multi-week novel movement-based intervention was effective in helping refugee youth improve movement competencies across all domains. PL can be seen as an important framework to help support people in becoming more active, and may be a way to help support newcomer youth gain exposure to a variety of opportunities to develop PL and sustained PA behaviors.

While there were significant increases found in movement competence, it is important to highlight that no significant changes in perceived competence, confidence, motivation, and knowledge and understanding were found. These findings are somewhat in contrast to a similar PL-based intervention focusing on university students (20), whereby significant increases in confidence, motivation and knowledge and understanding were evident without significant changes in movement competence. Current findings may be indicative of a group of youth faced with many changes during their transition into Canada. Participation within sport and other movement activities may have been limited during this time, and greater increases in movement competencies may be due to the opportunity to practice movement skills during this critical youth developmental period. Additionally, while there has been evidence to support the validity and reliability of the PLAY*self* tool (24), this study was the first to apply this with a newcomer population with limited English abilities. This may have impacted the ability to effectively capture change in these other core domains of PL. More importantly, more research in the future is needed to better understand PL assessments in diverse linguistic and cultural populations (17).

### Implications for practice

Young people migrating to a new country bring with them a diverse set of sport and fundamental movement skills influenced by their cultural backgrounds (25). As they integrate into their new environment, there is a promising opportunity to enhance their PL, given the adaptability and resilience often demonstrated by youth. It is crucial to acknowledge that preferences for sports and activities will varies across regions and countries due to distinct cultural preferences and historical influences (15, 26); therefore, exposing newcomers to a variety of movement skills that align with popular activities within their new community is essential for their successful integration and overall well-being. PL is considered an important antecedent to physical, social, and mental health and wellbeing outcomes through sustained physically active lifestyles (14), although PL research among immigrant and refugee populations remains scarce. While the current intervention emphasizes motor competence and sport-based activities, the concept of PL encompasses broader affective, cognitive, and motivational components that support lifelong engagement in active living (15). Future programming should embed opportunities for reflection, cultural relevance, and community connection to foster sustained motivation and confidence beyond the sport context. Efforts to introduce newcomers to movements that can be translated into popular activities, creating environmental contexts that can help to facilitate their integration into new communities (27), with more research to understand the impact of PL development on settlement outcomes needed.

Although the current study represents one of the first intervention studies targeting PL development among newcomer youth, there are several limitations that must be considered. First, the sample size for the study was relatively small with a gender imbalance and included a cohort of refugees predominantly coming from one country (Afghanistan), limiting the generalizability of these findings. Larger trials and more diverse samples of newcomers are needed. Second, the duration of the program was relatively short. For pragmatic reasons, the IPLAY program was being offered one time per week for 8 weeks may have been too short to impact all domains of PL. Future programs using novel movement skill development may consider more frequencies and longer program durations. Third, while validated measures were used in this study, it should be noted that there were language barriers for many of the participants and questions may have had difficulty with the interpretations of the questions and/or were perhaps not familiar with the contexts for which the questions were being asked. Future work that considers the cultural and linguistic adaptation is needed.

## Conclusions

In summary, the IPLAY intervention demonstrated some success in PL development among new refugee youths in Canada. Specifically, the program found significant increases in movement competences, which may be important towards participation in new activities during their transition into a new country. Future work needs to explore how to further target the other domains of PL, including confidence, motivation, and knowledge and understanding. This program provides an important foundation in PL development of newcomer youth, with the goal of having this population be able to develop and sustain healthy, active lifestyles during their transition to Canada.

## Data Availability

The raw data supporting the conclusions of this article will be made available by the authors, without undue reservation.
